# Photoinduced charge transfer in quasi-one-dimensional polymers in two-photon absorption†

**DOI:** 10.1039/d0ra06436j

**Published:** 2020-09-09

**Authors:** Pen-ji Yan, Xijiao Mu, Jun Dong, Mengtao Sun

**Affiliations:** College of Chemistry and Chemical Engineering, Key Laboratory of Hexi Corridor Resources Utilization of Gansu Universities, Hexi University Zhangye 734000 PR China; School of Mathematics and Physics, Advanced Innovation Center for Materials Genome Engineering, Beijing Key Laboratory for Magneto-Photoelectrical Composite and Interface Science, University of Science and Technology Beijing Beijing 100083 PR China mengtaosun@ustb.edu.cn; School of Electronic Engineering, Xi'an University of Posts and Telecommunications Xi'an 710121 China dongjun@xupt.edu.cn

## Abstract

In this work, we theoretically investigate the structure and the transition characteristics of one- (OPA) and two-photon absorption (TPA) spectra of different length neutral and charged thiophene polymers. The effects and regulation of different charges on photoinduced charge transfer are discovered and their physical mechanisms are explained. We find that both the OPA and TPA spectra undergo a sizeable redshift after the charge is injected into the polymer, and the redshift after the positive charge injection is excellent. The alternating charge transfer that occurs in a two-photon transition of a charged system is derived from the alternating distribution of charge (dipole moment) in the dynamics of the system. To study the gradual behavior of infinite polymers, we also theoretically calculated the optical properties and electronic structures of infinitely long thiophene polymers under different electrical charge injections by a one-dimensional periodic model. The redshift of the OPA and TPA spectra is found to be due to orbital energy level movement.

## Abstract 2

In this work, we theoretically investigate the structure and the transition characteristics of one- (OPA) and two-photon absorption (TPA) spectra of different length neutral and charged thiophene polymers

## Graphical abstract

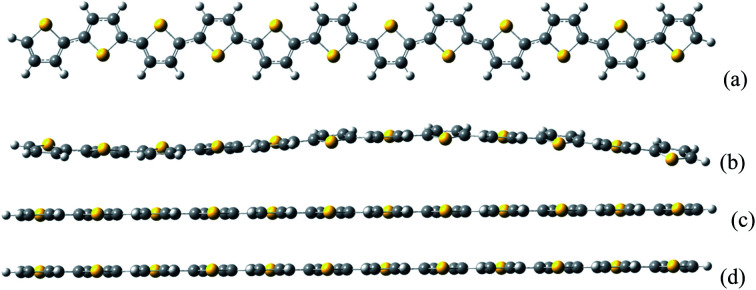

## 1. Introduction

In recent years, with the development of organic electronics,^1,2^ flexible electronic devices such as flexible screens and light-emitting devices (LEDs),^3,4^ dye-sensitized solar cells (DSCs),^5,6^ and the like have significantly been developed.^7–9^ Among them, polymers, predominantly conjugated polymers, are significant materials in organic electronics. Because of their good electron transport,^10^ light absorption and electroluminescence properties,^9,11^ the conjugated polymers play a key role in many regions of optoelectronics devices.^9^ Conjugated organic polymers can be easily modified to modify their electronic structure, which is different from traditional inorganic, silicon-based electronics. In many applications, unlike isolated neutral molecules, polymers are often in the presence of different electrical charge injections.^12^ The electron structure of the polymer changes after charge injection, so its optical properties also change greatly. This change cannot be ignored when designing and manufacturing devices. Therefore, it is necessary to study the structure of the polymer system in different electrical properties, the electronic wave function and the change of light absorption properties. Many applications of conjugated polymers in organic electronics, such as electron transport and optoelectronic devices,^13^ are inseparable from photoinduced charge transfer properties. The photoinduced charge transfer properties of polymers with different electrical charge injections have good research value. Two-photon absorption (TPA) is different from one-photon absorption (OPA) and is a nonlinear optical process.^14^ The TPA process can absorb relatively low energy photons to excite the system to a relatively high excited state.^15,16^ Therefore, TPA is often used in the biomedical field to observe fluorescence and ensure that samples are not damaged by high-energy lasers.^17–19^ In recent years, the application of TPA has been dramatically expanded. TPA can be applied not only in the field of biology, but also in materials science. TPA can excite a good two-photon excited fluorescence (TPEF) signal in a two-dimensional material to perform an exact optical imaging of the material.^20^ Therefore, it is also necessary to study the TPA characteristics of conjugated polymers which are very important in organic electronics. The traditional TPA calculation method is the quadratic response theory. This method is a good calculation of the TPA spectrum of the system. However, in order to analyze the two-step transition characteristics in the two-photon transition process well, the sum-of-states (SOS) based method is more convenient.^21^ This method can well analyze the charge transfer between the ground state and the intermediate state, the intermediate state and the final state during the two-step transition of TPA.^22^ This is very important for studying the charge transfer of polymer systems.

In this work, we theoretically studied nonlinear optical properties of thiophene polymers and oligomer models with different lengths and different charges. To simulate closer to the real situation, that is, to keep the electronic state multiplicity constant, the number of injected charges remains even. There are many studies on nonlinear optical properties of thiophene polymers in TPA,^23–25^ but few studies on their physical mechanisms, which can guide the synthesis on new nonlinear optical-electronic devices. The transition characteristics of OPA and TPA in isolated systems are analyzed. The optical properties and electronic structures of one-dimensional periodic thiophene polymers were theoretically analyzed by establishing an infinitely long periodic model. The regulation of charge on polymer OPA, TPA and other optical properties is summarized and explained and discussed theoretically.

## 2. Method

### 2.1 Quantum chemistry isolated system calculation

In order to investigate the effect of charge transport on the optical properties of polymers in the application scenario of optoelectronic devices, all the thiophene oligomers (*n* = 4, 8 and 12) geometries with different electrical charges are optimized by density functional theory (DFT)^26^ in combination with B3LYP functional,^27^ 6-311g* basis sets,^28^ SMD solvent model^29^ and DFT-D3 dispersion correction^30^ parameters with Gaussian 16 A.03 program.^31^ And based on the optimized structure, the time-dependent DFT (TDDFT),^32^ the CAM-B3LYP functional,^33^ SMD model and the 6-311g* basis sets are used to calculate and output all the configuration coefficients. By using the results of the above quantum chemical calculations, TPA spectra were calculated in combination with our TPA calculation program^22^ based on SOS method. The calculation results of this method are very close to the quadratic response theory.^34,35^ The atomic dipole moment corrected Hirshfeld (ADCH) charge,^36^ transition density matrix (TDM)^22,37^ with the color bar value from 0 to 1 and charge difference density (CDD) with isovalue of 0.005 are visualized by wave function analysis software Multiwfn-3.7 program^38^ and visualization software VMD.^39^

### 2.2 *Ab initio* molecular dynamic (AIMD) simulation

To analysis the molecular structure evolution of isolated thiophene oligomer after different charge injected, in this work, the 4 ps (picosecond) AIMD^40^ (NVT ensemble with 298 K) with the SMD solvent model and the berendsen thermostat^41^ are simulated by ORCA-4.2.1 program.^42^ The molecules structures is are first optimized and then carried out 12 000 steps AIMD processes with a step size of 0.5 fs (femtosecond). The total simulation time is 6 ps. To make the time equivalence of the subsequent wave function analysis, a wave function file is output every 10 steps processes. The ADCH charge and electron localization function (ELF)^43^ are analyzed and rendered by Multiwfn-3.7, VMD and our program.

### 2.3 First principles periodic calculation

In the periodic calculation, we have established an orthogonal lattice. And two thiophenes are the smallest repeating unit, and the direction of the periodic boundary is consistent with the length direction of the polymer. A vacuum layer of 15 angstroms was left in the other two directions. Calculations were performed using the QuantumATK-2018.06-SP1 software package.^44,45^ The atomic center basis set and the GGA-PBE functional^46,47^ are used in the calculations. Full optimization of the atomic geometry was performed until all components of the residual forces were less than 0.05 eV Å^−1^ and the total energy is converged within 10^−6^ eV. The *k*-mesh is 7 × 1 × 1 and the cut-off energy is 1200 eV. Using the same cut-off energy in the calculation of optical properties, the *k*-mesh is increased to 15 × 1 × 1, and the self-consistent field convergence limit is increased to 10^−8^ eV.

## 3. Results and discussion

### 3.1 Physical properties and AIMD processes at ground state

**Fig. 1:**
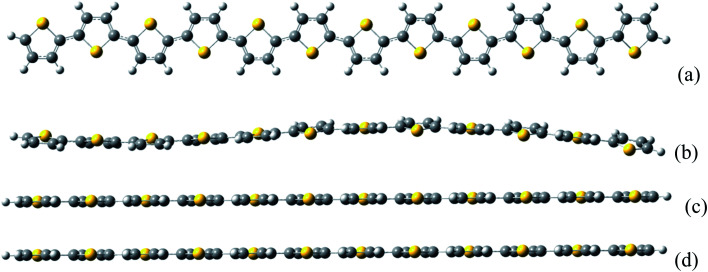
The thiophene oligomer molecular structures. (a) Top view of neutral thiophene oligomer. The (b–d) are side views of neutral, negative, and positive charged thiophene oligomer, respectively.

Fig. 1 demonstrates that the optimized molecular geometries for neutral and charge system. It is found that for the neutral thiophene oligomer, the structure is not perfectly flat; while for the charge system, the oligomer geometry is perfect flat.

**Fig. 2:**
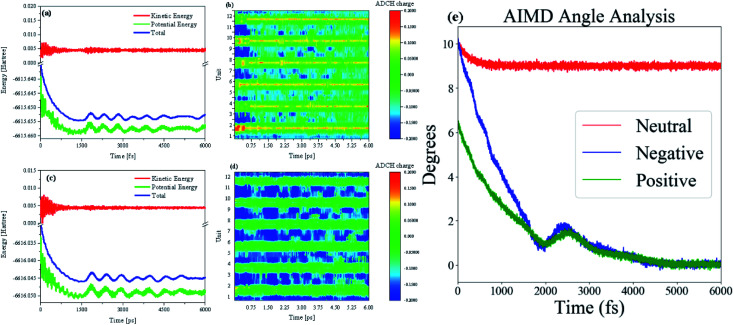
The AIMD energy details of positive (a and c) negative thiophene oligomer. (b and d)The difference of ADCH charge in the AIMD process of positive and thiophene oligomer, respectively. (e) The angle analysis between two direction vectors of thiophene oligomers in AIMD process.

To dynamically analyze structural changes in charged oligomers, we used molecular dynamics (MD) methods to observe the behavior of the oligomer after charge injection. Besides, we need to monitor and analyze the changes in the wave function in real-time during the MD process to explore the physical principles of this change. So the AIMD method is used to study this process. Throughout the AIMD process, the first 250 fs process is a process in which the charged oligomer energy tends to be extremely small (Fig. 2(a) and (c)), and the thermostat plays an essential role in this process. Therefore, the half-times is about 125 fs. As shown in Fig. 2(a) and (c), after reaching the first minimum energy value, the energy begins to rise again, and this energy fluctuation continues until 2 ps to make the oligomer straight, see the middle of Movies S1 and S2 in ESI.† But just after reaching the minimum value, the process is not stable and will fluctuate until it reaches true straightness and stability after 3 ps (for the situation of negative charge injection, the time of straight and stability is 3.5 ps), see the middle of Movies S1 and S2 in ESI.† After the charge is injected into the neutral oligomer system, the type of charge has little effect on the ELF. However, after negative charge injection, the structure and ELF relaxation rate is slightly slower (Movie S2†), and the amplitude of energy fluctuation is also larger than that of positive charge injection, see Fig. 2(c). The size of the ELF isosurface can characterize the degree of conjugation. Therefore, it can be found that the twist of the thiophene oligomer is directly proportional to the degree of conjugation. This is because during the injection of negative charge (adding electrons), more electrons are introduced into the system, which increases the vibration damping of the structure at a certain temperature. The physical mechanism can be interpreted as below several reasons. After charge injection, the curved thiophene conjugated long chain begins to relax. As the long chain relaxes from the bend to the straight, the value of the ELF becomes smaller and its isosurface becomes larger. This process shows that the injection of charge leads to a decrease in electron localization and an increase in electron mobility, see Fig. S1(a) in ESI.† The abscissa is the number of optimization steps, and the ordinate is the ADCH charge. This shows that after the electron injection, the charges at both ends are not equal, and the coulomb interaction between the charges will promote the flattening of the long chain.

After charge injection, the distribution of charge on the long chain is on the edge. Whether it is positive charge injection or negative charge injection, both ends of the charge will be transferred from the middle. The distribution of charge on the cell is such that the absolute value at both ends of the long chain becomes smaller and the absolute value at the center becomes larger, please refer to Fig. S1(b andc) in the ESI.† Because the definition of the charge population is the Hirshfeld charge corrected by the atomic dipole moment. The correction charges are expected to be distributed only around atoms neighboring to A, this could be realized by minimizing function *F*:
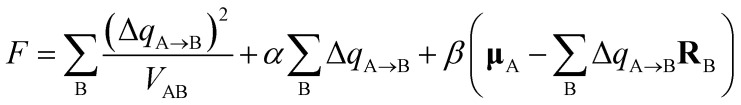
where the Δ*q*_A→B_ is the exchange of charge from A to B atom and the **μ**_A_ is the dipole moment of A atom. The **R**_B_ is the position vector of B atom and the *α* and *β* is the Lagrangian multipliers used to satisfy the two constraint conditions. This definition can reflect the dynamic process of charge changes. Therefore, this shows that the structure changes from bending to straightness due to dipole moment changes during charge transport. The ADCH charge is a Hirshfeld charge that has been corrected by atomic dipole moment. Although the definition of this charge is inconsistent with chemical intuition, it can perfectly fit the molecular permanent dipole moment. This is particularly important to explain the change of dipole moment in the TPA process. On this basis, we analyzed the changes in ADCH charge during the AIMD process, see Fig. 2(b) and (d). It can be seen from the figure that in the oligomer, the ADCH charges of different units are substantially opposite to each other. That is, the stripes of different colors in the figure. This alternating change creates a non-zero dipole moment between the elements. At the beginning of the simulation, the charge and the internal difference between the two ends of the oligomers are large, thus promoting the straightening of the long chain. As the simulation progresses, the oligomer gradually flattens and the ADCH charge on the cell tends to balance, so the dipole moments between the cells cancel each other out, leaving the system in equilibrium. Overall, the charge change of ADCH is different from that of positive charge after negative charge injection. Since the entire system is in a negative state, it is difficult to find a positive ADCH charge region in Fig. 2(d). However, the law of alternating charge changes is still as described above. The ADCH charge distribution in the neutral system is relatively smooth, see Fig. S2 in ESI.† Due to the asymmetry of the charge distribution, the thiophene oligomer after charge injection tends to be straight. We have defined the twist angle of the thiophene oligomer. This angle is the angle between the vector formed by the middle atom and the edge atom. Use this angle to observe the change in angle during the AIMD process, see Fig. 2(e). It can be found from the figure that with the progress of the simulation, the included angle of the charged system decreases continuously, and finally reaches zero degrees. The angle of the neutral system is relatively large, and the simulation has a linear downward trend, but it is basically maintained at about 9°, and is significantly larger than the charged system.

### 3.2 Optical absorption spectra

**Fig. 3:**
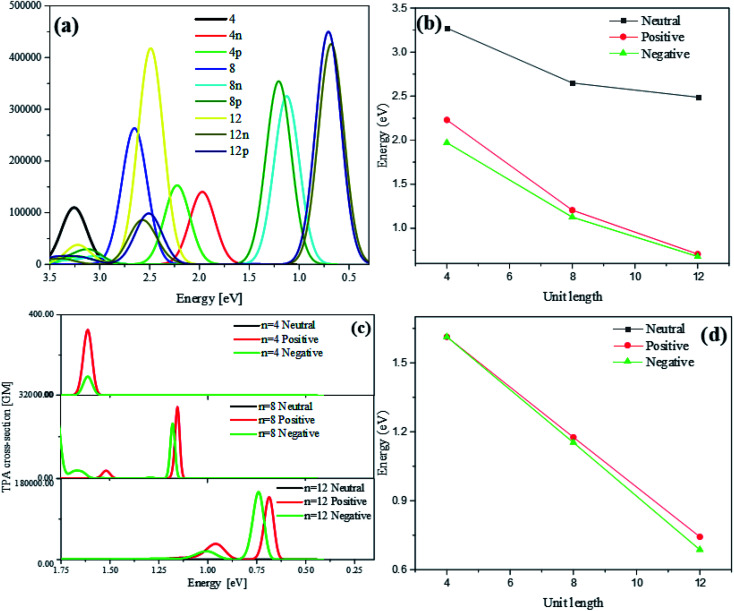
The OPA (a) and TPA (c) spectra of different length thiophene oligomer. The change of excitation energy of neutral, positive and negative system strongest absorption peaks in S_1_ of OPA (b) and S_2_ of TPA (d).

Optical absorption spectra of neutral and charged thiophene oligomer with different length (*n* = 4, 8, 12) in one photon absorption (OPA) and TPA are calculated, which can be seen from Fig. 3(a). With the different number of units (*n* = 4, 8, 12), for neutral and charged thiophene oligomer in OPA in Fig. 3(b), it is found with the increase of the oligomer lengths, the excitation energy is gradually red-shifted. For the thiophene oligomer with different numbers of units, the excitation energies for the charge system are significantly less than that of the neutral system, and with the increase of length of thiophene, the energy difference between neutral and charged system at the same unit is larger.

Fig. 3(c) is the optical absorption spectra of neutral and charged thiophene oligomer in TPA. It is found that they are the same tendency for the excitation energy: with the increasing of the unit of thiophene, the excitation energy is gradually red shifted, see Fig. 3(d). Note that the absorption peak for neutral system in TPA cannot be observed. The reasons may be that the absorption peak for the neutral system is too weak to be observed, and/or the excitation energy is too large to be observed in the region in Fig. 3(c) and (d). Fig. S3(a) and (b) that in ESI† demonstrate that reasons, resulting from the above two proposed reasons.

### 3.3 Photoinduced charge transfer in OPA

**Fig. 4:**
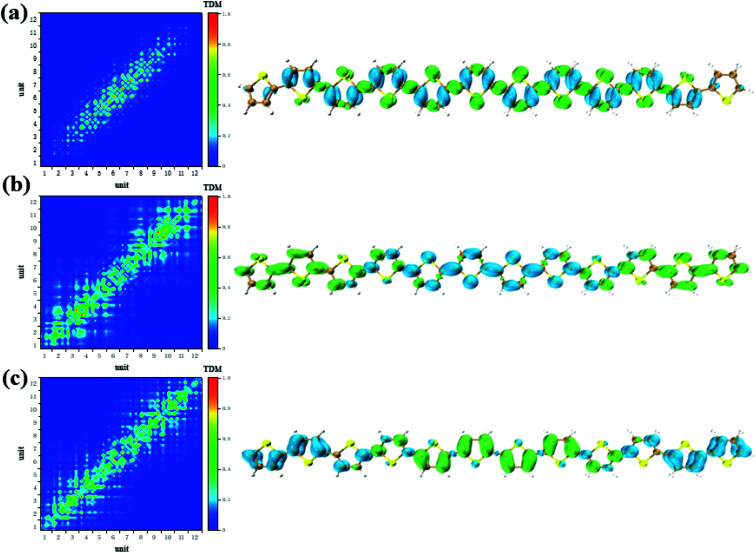
The neutral (a), positive (b) and negative (c) system S_1_ transition characteristic (TDM and CDD) of thiophene oligomer (*n* = 12) in OPA.

CDD and electron–hole pair analysis can intuitively and quickly analyze the charge transfer phenomenon in the process of electron transition in conjugated systems.^47^ Now, we study the optical properties of photoinduced charge transfer in OPA. Due to the number of units = 12 is the largest system, so the optical properties of photoinduced charge transfer in OPA is employed. Fig. 4(a) demonstrate the electron–hole coherence and visualization of charge transfer in neutral thiophene in OPA. The transition density matrix reveals that central excitation of electron–hole is strongest, and two edge units are almost not excited. The excitation length is within three units, which means that electron–hole coherence within three units. The CDD diagram of Fig. 4(a) reveal the orientation and results of photoinduced charge transfer in thiophene oligomer (*n* = 12), it is found the electron and hole can only be delocalized to their neighboring units, which is the typical Frenkel excitations. When the thiophene oligomer is injected by charge, the transition characteristic undergoes tremendous changes. When a positive charge is injected, charge transfer excitation from the center to both ends appears across the thiophene oligomer, see Fig. 4(b). The results presented by TDM are not only the charge transfer from the center to the ends but also the local excitation of the oligomer. This is because the excess charge exhibits stronger delocalization after positive charge injection. Therefore, only charge transfer excitation can be seen in the CDD map, see the right part of Fig. 4(b). After the negative charge injection, it also exhibits a strong charge transfer excitation. However, unlike the case of positive charge injection, the orientation of charge transfer is exactly the opposite of charge transfer from the two ends to the center. According to the analysis in Fig. 2(b), the reason for this phenomenon is that the extra injected charges are concentrated in the middle after the structure tends to balance. However, after the system is excited by photons, this balance of charge is broken, causing a strong charge transfer. When the length of the oligomer is different, this phenomenon still exists. After charge injection, the electron excitation will change from local excitation to charge transfer excitation, see Fig. S4 in ESI.† However, this phenomenon is not apparent when the oligomer is short (Fig. S4(a–c) in ESI†), and becomes apparent when the length reaches *n* = 8.

### 3.4 Photoinduced charge transfer in TPA

**Fig. 5:**
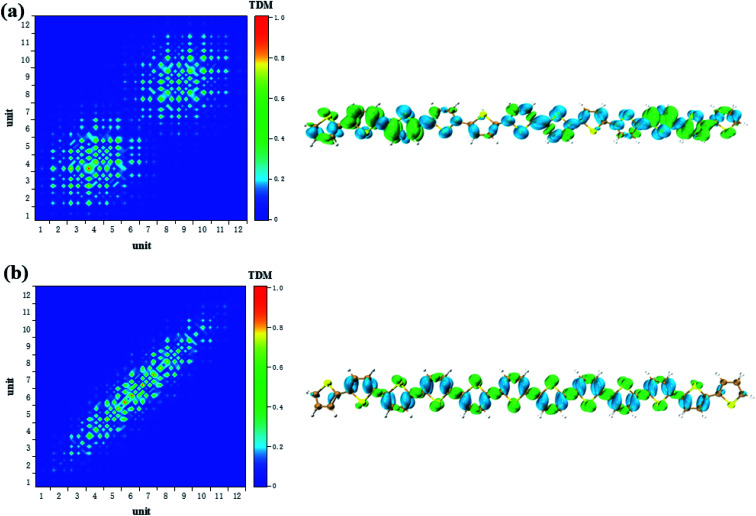
The S_1_ → S_2_ (a) and S_0_ → S_1_ (b) transition characteristic of neutral thiophene oligomer in two-step transition model of TPA.

**Fig. 6:**
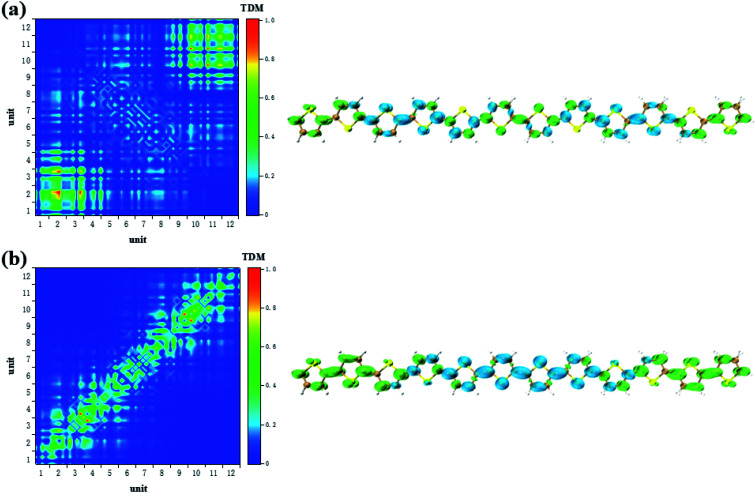
The S_1_ → S_2_ (a) and S_0_ → S_1_ (b) transition characteristic of negative charged thiophene oligomer in two-step transition model of TPA.

**Fig. 7:**
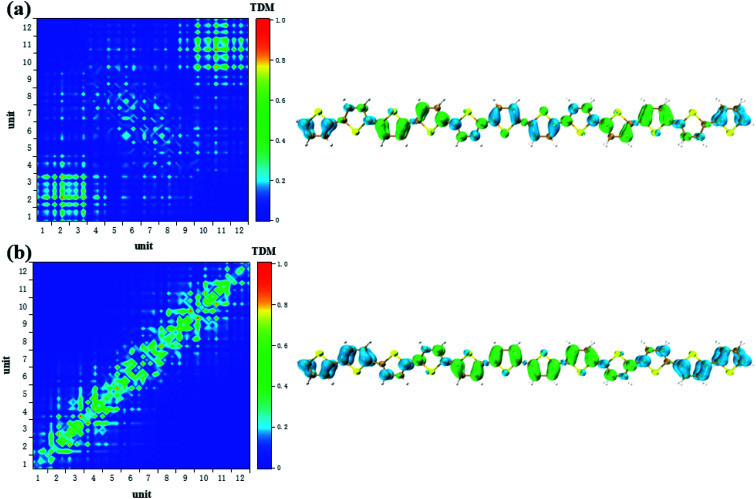
The S_1_ → S_2_ (a) and S_0_ → S_1_ (b) transition characteristic of positive charged thiophene oligomer in two-step transition model of TPA.

According to the analysis in Fig. 3, the strongest peak of TPA is the S_2_. Through the analysis of S_2_, it is found that this excited state is mainly contributed by a two-step transition. And the intermediate state in the two-step transition model is S_1_. Therefore, the first transition in the two-photon transition is from the S_0_ to the S_1_, and the second transition is a transition from the S_1_ to the S_2._ According to the discussion in the previous section, after the system with additional charges, the transition from the ground state to the S_1_ changes from the local Frenkel excitation to the charge transfer excitation. Moreover, charge transfer in different orientations are exhibited depending on the difference in charge. The second step in the two-photon transition is also affected by the extra charge. Firstly, when there is no charge, that is, a neutral oligomer, the second step transition shows locality. But this localized excitation is present on both sides of the oligomer, see Fig. 5(a). Secondly, after the positive charges are added to the system, the second step transition also shows a significant charge transfer. Moreover, unlike neutral systems, the units at both ends of the oligomer also have a small electron density, see Fig. 6(a and b). In OPA, after the charge is added, the transition characteristic is the charge transfer excitation between the center and the edge of the oligomer. However, in the second step of TPA, this charge transfer is an alternating charge transfer in units of two thiophenes, and the edges of the oligomer are electrons density, see Fig. 6(a). Thirdly, after the negative charge is added to the system, the second step of the TPA transition is also an alternating charge transfer excitation, but the difference is that the oligomer edges are holes density, see Fig. 7(a and b). This alternating charge transfer is derived from the alternating distribution of the ADCH charge and dipole moments embodied in the AIMD process, see Fig. 2(b) and (d). Finally, it is not difficult to find that after the negative charge injection, the second step of the TPA transition is the transition between the p orbital and the π orbital, that is, the p–π* transition, and the positive charge injection is followed by the transition between the p orbitals. This is because the so-called negative charge injection is a process of adding electrons, and positive charge injection is a process of taking out electrons. Thus, for molecular systems, negative charge injection increases the frontier orbit, so the transition occurs between the higher energy p-orbitals and the π anti-bonded orbitals. Positive charge injection reduces the frontier orbit, and the transition occurs between the lower energy p-orbitals. This phenomenon is more clearly reflected in the analysis of infinitely thiophene oligomers. When the lengths of the oligomers are different, the transition characteristics of TPA change with the same charge as discussed above, that is, there are alternating charge transfer of units and the type of charge determines the orientation of charge transfer, see Fig. S5 and S6 in ESI.† The charge on the charged thiophene oligomer is periodically distributed during the AIMD process, which also leads to the periodic transfer of photon-induced charge transfer.

### 3.5 First-principles periodic calculations for 1D infinite thiophene polymer with different additional charges

**Fig. 8:**
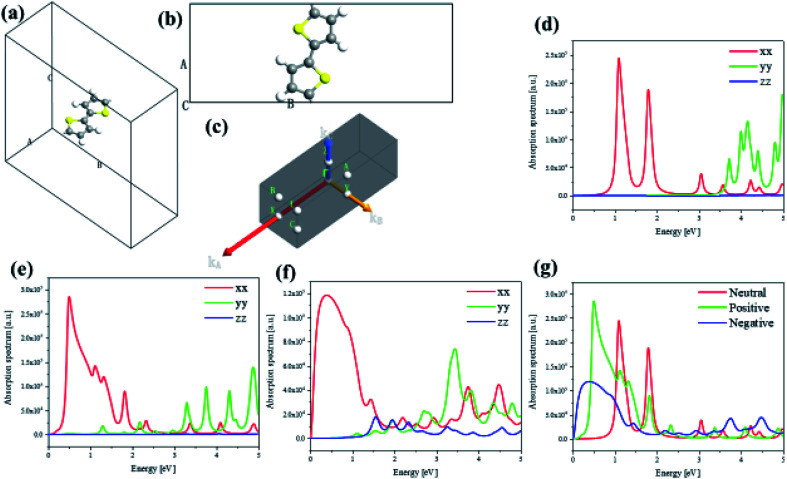
The periodic cell structure main view (a) and side view (b) of 1D infinite length thiophene chain and its Brillouin zone (c). Electron absorption spectra of 1D infinite length thiophene neutral (d) chain and with positive (e) or negative (f) charge injection, and their comparative result (g).

To investigate the transition and optical properties of polymer with different additional charges, we study on the 1D infinitely long thiophene chain with first-principles calculation. Periodic first-principles calculations can well reflect the electronic properties of infinite polymers. For the one-dimensional infinite system, a periodic model is used, see Fig. 8(a). As shown in Fig. 8(b), to represent the features of the 1D long chain in the calculation, a vacuum layer of 10 angstroms in length is established in the B and C directions of the crystal lattice, and the A direction is a periodic boundary, see Fig. 8(b). Since this structure is input to the triclinic system, the distribution of the Brillouin zone and the high symmetry point is set as shown in Fig. 8(c). On this basis, we have analyzed and studied the optical properties and electronic structure of the system. Firstly, Fig. 8(d–f) shows the anisotropic absorption spectra of the system with neutral, positive and negative charge injections, respectively. Since the system is a one-dimensional quantum wire, only the absorption behavior in the *xx* direction has practical significance. It can be seen from the figure that after the charge injection, there is strong absorption in the low energy region. Before the charge injection, that is, the neutral system, there are two distinct absorption peaks at positions around 1 eV and 2 eV. However, after charge injection, there are three absorption peaks in the low energy region (whether positive or negative charge injection). The position of the strong absorption peak also undergoes different degrees of red shift, and the negative charge injection is more red shift. However, the absorption intensity becomes smaller when the negative charge is injected, and the absorption intensity is the largest when the positive charge is injected, see Fig. 8(g).

**Fig. 9:**
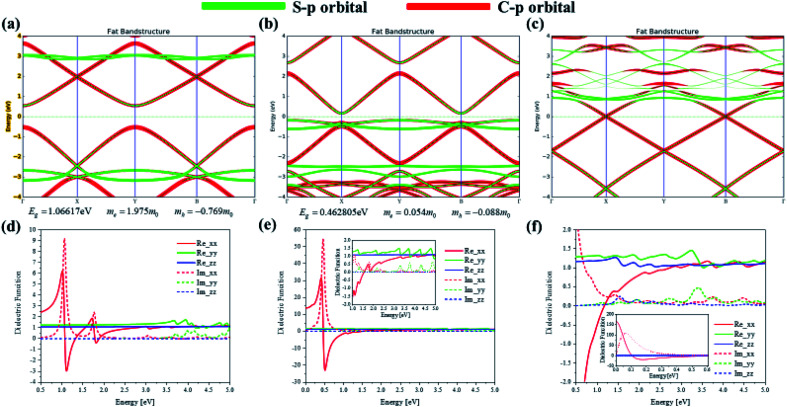
Projected energy band structure and anisotropic dielectric function of 1D infinite length neutral (a and d) thiophene chain and with positive (b and e) or negative (c and f) charge injection.

To deeply study the reasons for the above changes in the system after charge injection, the band structure and anisotropic dielectric function of the system are calculated under different conditions (charge injection). In order to deeply study the reasons for the above changes in the system after charge injection, the projected energy band structure and anisotropic dielectric function of the system are calculated under different conditions. The Fermi level of the neutral system is centered in the two energy bands, so the system exhibits the properties of a semiconductor with a band gap of 1.07 eV, see Fig. 9(a). It can be found that after the positive charge injection, the band gap becomes narrower and becomes 0.46 eV, see Fig. 9(b). This is also the main reason for the red shift of the absorption spectrum. However, the band gap appears to be small. In fact, after the positive charge injection, the electrons of frontier orbital are removed, so the original valence band becomes a conduction band. In other words, the equivalent of the HOMO becomes LUMO, and the original HOMO-1 becomes HOMO. Therefore, the band gap becomes smaller because the band can move upward as a whole. Moreover, after positive charge injection, that is, after electron loss, the effective mass of electrons decreases from 1.975 to 0.054, and the effective mass of holes increases from −0.769 to −0.088. The size of the mobility can be studied by the size of the effective mass, and the effective mass can be calculated by the band curvature. This indicates the electron's and hole's localization properties is reduction and the mobility is increased. This will help photoinduced charge transfer. On the other hand, after the negative charge injection, the system Fermi level passes through the energy band, and the system has a part of the metal properties, see Fig. 9(c). Therefore, the absorption peak will be further red-shifted. This metallicity also causes the better electron transport properties and enhances photoinduced charge transfer. Next, the anisotropic dielectric functions are calculated, which are the core parameters of the optical properties. It can be seen from Fig. 9(d) and (e) that the neutral system has two distinct imaginary peaks, which correspond to the absorption spectra. After positive charge injection, the real and imaginary parts of the dielectric function (*xx* direction) increase significantly. At the position where the imaginary maximum is located, the real part of the dielectric function has a negative value. This shows that this system has potential for surface plasmon applications. On the other hand, after negative charge injection, the system has metallic properties. The real part of the dielectric function of the low-energy region has a large negative value, and there is also a strong imaginary part, see the insert picture of Fig. 9(f). The electronic structure and optical properties of one-dimensional infinite length (polymer) and finite length system (oligomer) are different. This is because the wave function of an oligomer is similar to an isolated molecule. The length of a single thiophene unit is only 4.46 Å, while the length of a 12-unit thiophene chain is only 5.35 nm. Compared with polymers, the optical behavior of oligomers is closer to the quantum dot behavior. Although the lattice of an infinitely long system has only the length of two thiophenes, the results of this section and the discussion show the properties of a sufficiently long 1D quantum wire due to the periodic boundary in the direction A of Fig. 8(b). Therefore, there are similarities and differences properties between the 1D infinite and the isolated finite-length system. The same is that electron mobility are significantly increased after charge injection, enhancing photoinduced charge transfer. The difference is that the difference in optical absorption and the low energy region dielectric function show significant surface plasmon properties. Because in this region, the real part of the dielectric function has negative values, and the imaginary part is greater than zero.

## 4. Conclusion

In this work, we first establish an isolated system model to theoretically study the absorption spectra and transition characteristics of OPA and TPA after different lengths of thiophene polymers carrying different charges. The charge has a relatively large effect on the structure of the polymer. From neutral to charged systems, the polymer structure becomes straight from bending. After charge injection, the local excitation of OPA is induced into charge transfer excitation, and the direction of charge transfer varies depending on the charge. The localized excitation of the second step transition in TPA also becomes an alternating charge transfer in units of two thiophenes after charge injection. According to AIMD results and animations, we can see that there is a significant correlation between charge injection and oligomer flattening. This phenomenon is due to the fact that the distribution of ADCH charge and dipole moment also has a strong periodic alternating distribution during AIMD. According to the calculation of the AIMD process, the curved oligomers have an alternating distribution of charges after charge injection, which directly leads to the alternating distribution of electrons and holes in the OPA and TPA processes. The orientations of charge transfer are also charge dependent in TPA. Secondly, we established a one-dimensional infinitely long thiophene polymer system and calculated the optical properties and band structure of the periodic charged system. The reason for the red shift of the OPA and TPA absorption peaks is the movement of the band. There is also a clear manifestation in the anisotropy dielectric function, especially along the orientation of the polymer. In summary, the addition of charge to the polymer has a strong regulatory effect on OPA and TPA transitions. This will have great application potential in organic electronics and polymer optoelectronic devices. In addition, this multi-scale computational simulation method (time scale and periodic scale) provides new possibilities for the study of new physical and chemical phenomena.

## The data availability statement

The data that supports the findings of this study are available within the article and its ESI material.†

## Conflicts of interest

There are no conflicts to declare.

## Supplementary Material

RA-010-D0RA06436J-s001

RA-010-D0RA06436J-s002

RA-010-D0RA06436J-s003
