# Supplementary material for: Photoinduced charge transfer in quasi-one-dimensional polymers in two-photon absorption
Source: RSC Adv. 2020 Sep 9;10(55):33288–98. doi: 10.1039/d0ra06436j (PMC9056739; doi:10.1039/d0ra06436j)
Supplement: RA-010-D0RA06436J-s003 [file RA-010-D0RA06436J-s003.pdf]

Electronic Supplementary Material (ESI) for RSC Advances.
This journal is © The Royal Society of Chemistry 2020

Supporting Information

Photoinduced Charge Transfer in Quasi-one-dimensional Polymers in Two-Photon Absorption

Pen-ji Yan,[1,+] *Xijiao Mu,*[2,+] *Jun Dong,*[3,*] *Mengtao Sun*[2,*]

1. College of Chemistry and Chemical Engineering, Key Laboratory of Hexi Corridor Resources Utilization of Gansu Universities, Hexi University, Zhangye 734000, PR China

2. School of Mathematics and Physics, Advanced Innovation Center for Materials Genome Engineering, Beijing Key Laboratory for Magneto-Photoelectrical Composite and Interface Science, University of Science and Technology Beijing, Beijing 100083, PR China

3. School of Electronic Engineering, Xi'an University of Posts and Telecommunications, Xi'an 710121, China

* Corresponding Author. E-mail: mengtaosun@ustb.edu.cn (M. T. Sun) and dongjun@xupt.edu.cn (J. Dong).

[+] Contributed Equally.

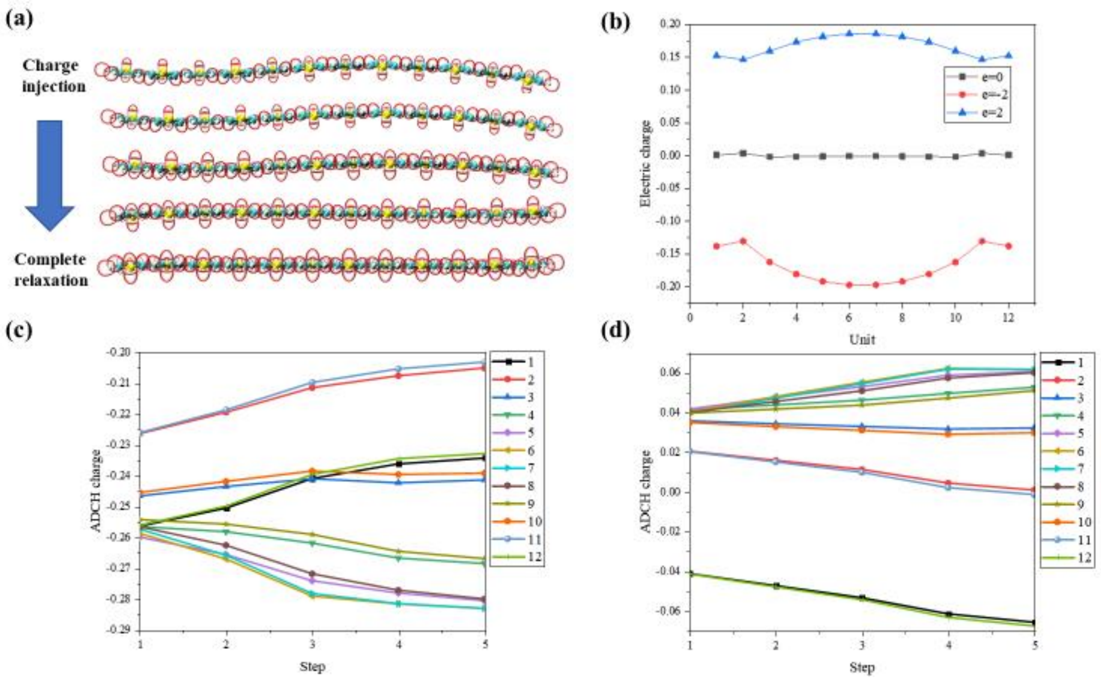

Figure S1. The ELF changes (a) of key steps, the Muliken population units distribution (b) and the ADCH charge changes of key steps in negative (c) and positive (d) thiophene polymer, respectively.

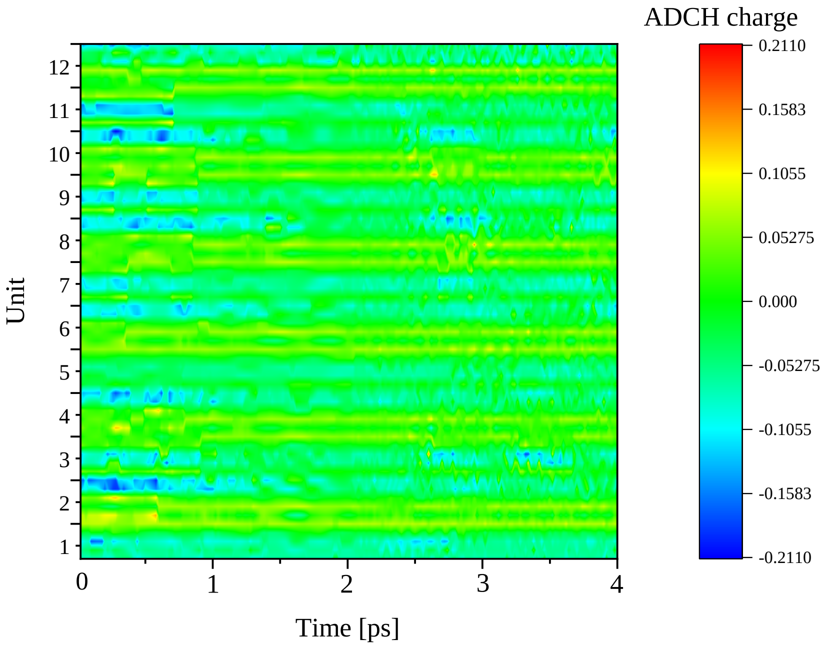

Figure S2. The difference of ADCH charge in the AIMD process of neutral thiophene oligomer.

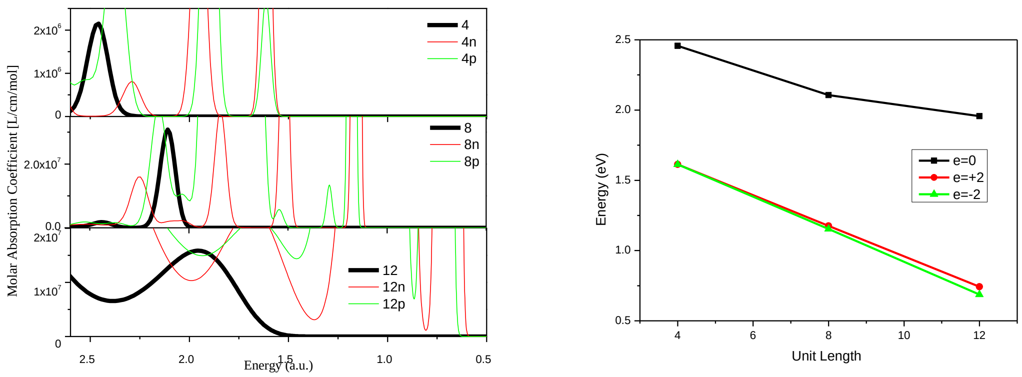

Figure S3. The TPA spectra of different length of neutral and charged thiophene polymer (a) and the relationship of unit length of thiophene polymer and excitation energy of main absorption peak (b).

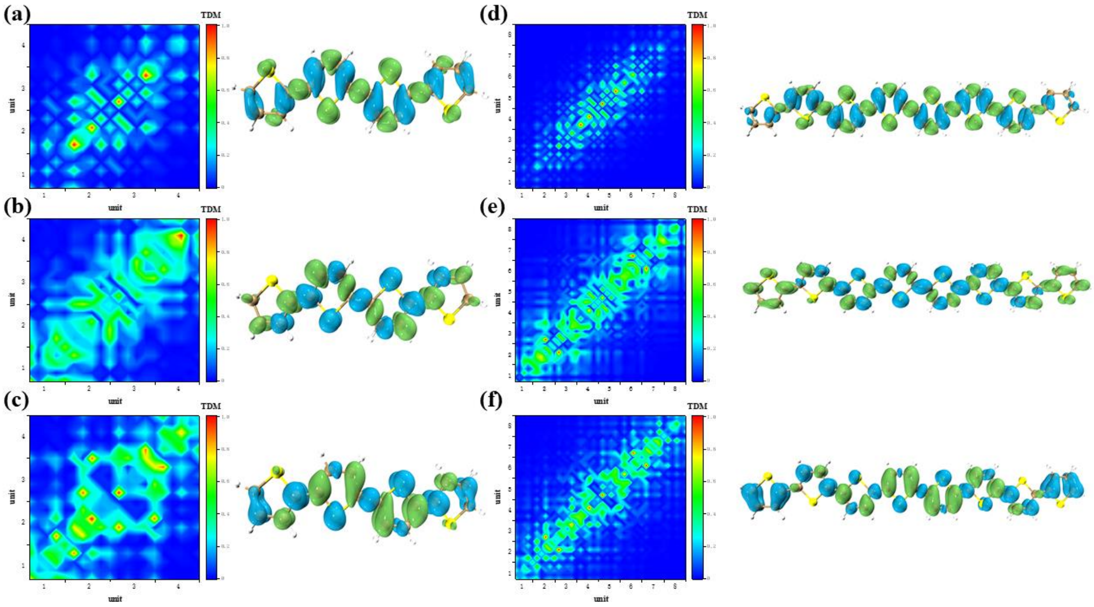

Figure S4. The OPA transition characteristic of neutral (a, d), positive (b, e) and negative (c, f) thiophene polymer (n=4 and n =8), respectively.

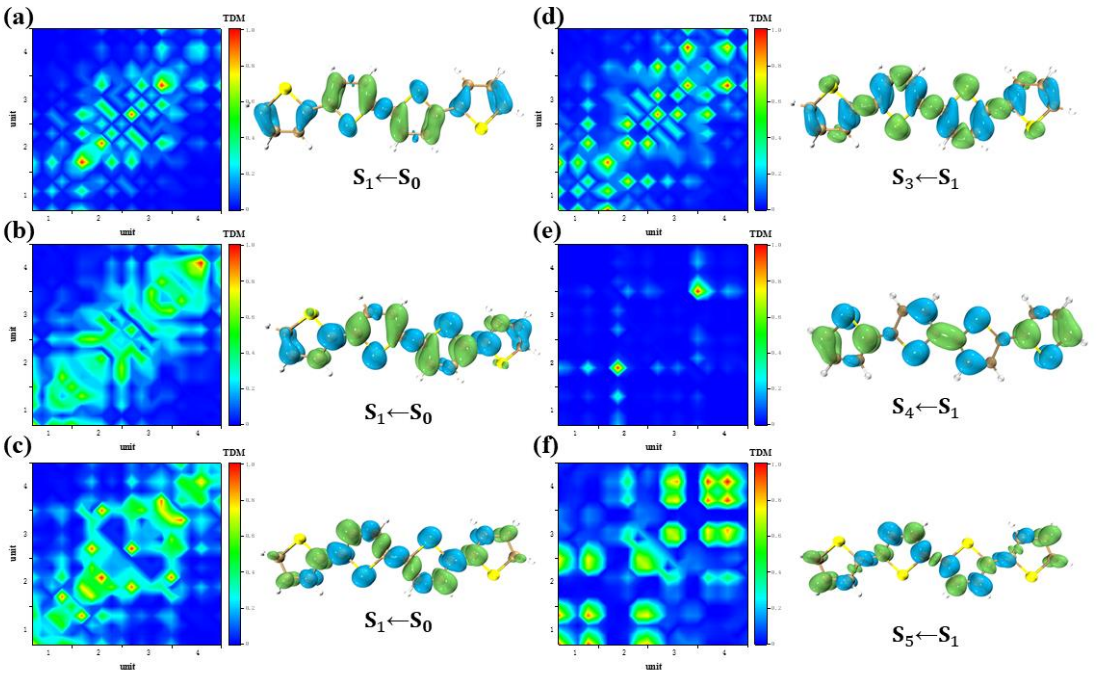

Figure S5. The first and second step transition characteristic in TPA of neutral (a, d), positive (b, e) and negative (c, f) thiophene polymer (n=4), respectively.

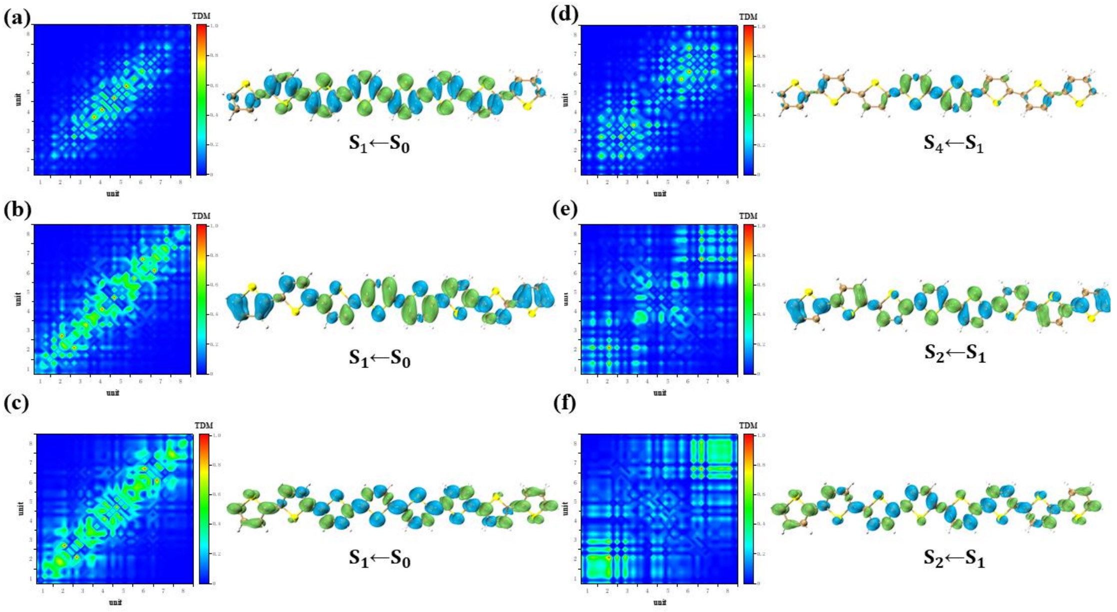

Figure S6. The first and second step transition characteristic in TPA of neutral (a, d), positive (b, e) and negative (c, f) thiophene polymer (n=8), respectively.
